# Eliminating ghost workers and optimizing resources to strengthen Community Health Worker programs in sub-Saharan Africa

**DOI:** 10.1371/journal.pmed.1004929

**Published:** 2026-02-17

**Authors:** Temesgen Ayehu, Josiah F. Joekai, Mulbah K. Yorgbor, Edleen T. Clark, George P. Jacobs, Christine E. Brooks-Jarrett, S. Olasford Wiah, Sagnon Fatoumata, Moussa Dialo, Loue Dékwinzou Frederic, Brima O. Kamara, Christine E. E. Williams, Mohamed S. T. Kamara, Matthew Beckhio, Adamou A. Marcelin, Noella Claire Mbrenga, Bawene Edouard, Sebakunzi Theirry, Hezagira Emery, Theophile Dushime, Hudad Barry, Haileyesus Getahun

**Affiliations:** 1 Health Development Partnership for Africa and the Caribbean, Kigali, Rwanda; 2 Civil Service Agency, Monrovia, Liberia; 3 Ministry of Health, Monrovia, Liberia; 4 Ministry of Civil Service, Labor and Social Protection, Ouagadougou, Burkina Faso; 5 Ministry of Health, Ouagadougou, Burkina Faso; 6 Ministry of Health, Freetown, Sierra Leone; 7 Ministry of Public Administration and Political Affairs, Freetown, Sierra Leone; 8 Ministry of Health and Population, Bangui, Central African Republic; 9 Ministry of Public Service and Administrative Reform, Bangui, Central African Republic; 10 Ministry of Health, Kigali, Rwanda; 11 Rwanda Biomedical Center, Kigali, Rwanda

## Abstract

Although community health workers (CHWs) play a vital role in filling health workforce gaps, they remain inadequately compensated due to insufficient domestic financing. In this Policy Forum article, Temesgen Ayehu and colleagues discuss how elimination of ghost workers and healthcare reform in Sub-Saharan Africa can release funds to properly pay and integrate CHWs into civil service programs.

**Author summary**

- A critical shortage of health workforce in sub-Saharan Africa (SSA) continues to impede progress towards Universal Health Coverage (UHC). This deficit is compounded by systemic inefficiencies, including the persistence of ghost workers, high absenteeism, and suboptimal performance that drain scarce public resources.
- Although Community Health Workers (CHWs) play a vital role in filling health workforce gaps and expanding access to essential health services, they remain inadequately compensated due to insufficient domestic financing.
- Eliminating ghost workers, as demonstrated in several SSA countries, can unlock resources to reinvest in frontline health workers, including CHWs. We argue that close collaboration between Ministries of Health and Civil Service agencies, with effective and comprehensive civil service reforms, will help to address human health workforce challenges.
- The newly established Health and Public Service Network of Africa (HaPSNA) provides a critical platform for collaboration between Civil Service Agencies and Ministries of Health, with the goal of improving efficiency and accountability in the health sector. By addressing persistent challenges, such as the prevalence of ghost workers and weak workforce management, the network seeks to improve governance and optimize the use of limited resources through South–South partnerships and peer learning.
- HaPSNA has developed a Community Health Program Maturity Matrix and Index to enable countries to self-assess the extent to which community health programs are integrated into primary healthcare and civil service systems, and to identify priority areas for improvement.

## Introduction

An adequate, competent, and motivated health workforce is fundamental to achieving Universal Health Coverage (UHC) and improving population health [1]. However, a 6.1 million health workforce shortage is projected for sub-Saharan Africa (SSA) by 2030, severely interfering with access to essential health services and impeding progress towards UHC [2]. Community Health Workers (CHWs) play a significant role in mitigating these workforce shortages and delivering primary care, particularly in underserved and rural communities [3]. CHWs deliver essential health, social, and community services to underserved populations in low-income settings [4], providing a continuum of preventive, promotive, and curative services across reproductive, maternal, newborn, and child health, as well as communicable and non-communicable diseases, thereby advancing progress towards UHC [5]. CHWs have significantly improved health outcomes, including a reduction in child and maternal mortality, and enhanced the care for malaria, tuberculosis, and HIV [4–7].

The positive impact of CHWs is substantially increased when they receive fair compensation, opportunities for skill development, and ongoing training support [8]. Despite their vital contributions, only about half of CHWs delivering government-supported services in low- and middle-income countries (LMICs) are salaried, with the remainder dependent on stipends, in-kind support, or unpaid voluntary work [9]. Financial constraints driven by limited fiscal space [10] and tight public budgets [11], competing priorities, and unpredictability of donor funding [10] have left many governments unwilling or unable to ensure sustainable employment for CHWs [12]. Therefore, CHWs frequently serve as volunteers or rely on ad hoc donor-funded stipends, leaving them little job security, inadequate pay, and few opportunities for career advancement [13].

**Fig 1:**
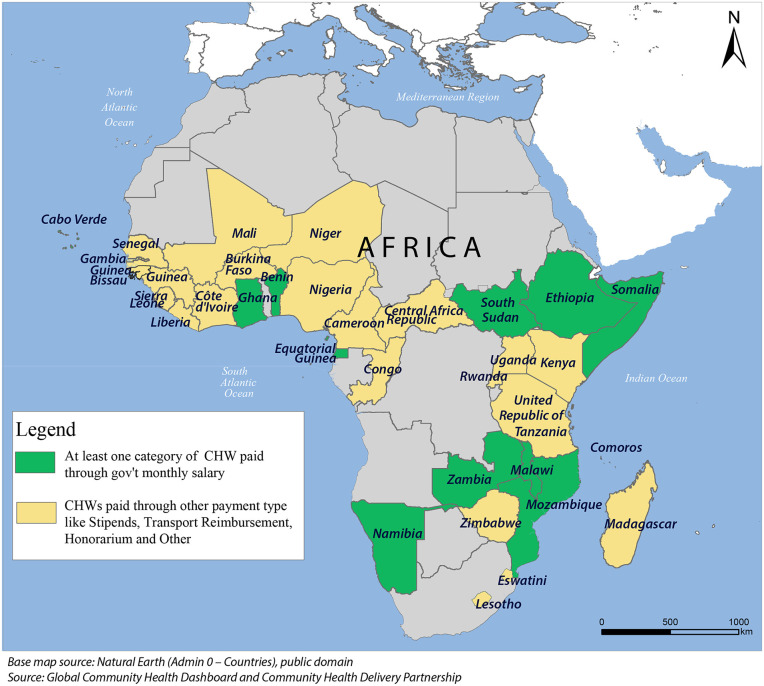
Types of financial remuneration of CHWs in Africa.

In many LMICs, essential frontline workers remain unpaid, unemployed, or underpaid, while paradoxically, public funds are directed towards payment of “ghost workers,” individuals who are fictitious, deceased, or absent from duty [14,15]. These contradictions expose systemic dysfunctions that perpetuate inefficiencies, including payroll fraud, weak human resource systems, and inadequate accountability mechanisms [14,16]. Ghost workers in human resources for health represent a global challenge [16]. In Africa, the problem of ghost workers stems largely from poor governance and corruption, creating a double injustice in which governments claim they cannot afford to pay real health workers while scarce resources are siphoned to fictitious employees who contribute nothing [17]. In SSA, among CHWs paid through financial remuneration, only 10 countries provide government salaries (Fig 1) [18]. Failure to resolve this paradox undermines health systems and leaves communities and vulnerable populations without access to healthcare. Such systemic bottlenecks limit the World Health Organization (WHO) African Region’s health system to only 77% of its potential [19]. This further fuels health inequities as marginalized populations are left without access to basic services [20].

## Unsustainable financing for CHW programs in Africa

In SSA, CHW programs remain heavily donor dependent, with external partners financing about 60% of the 1 billion USD invested, while governments contribute the remaining 40% [21]. According to the Community Health Dashboard maintained by the Community Health Delivery Partnership, as of December 2024, 41 African countries received donor support for CHW programs, including strengthening supply chain systems, developing strategies and policies, supervision, training, capacity building, and providing remuneration or stipends for CHWs [18]. For example, 22 African countries received funding from USAID and other U.S. government agencies, including PEPFAR, PMI, and CDC [18].

Recent abrupt cuts in foreign aid from major donors, including USAID and other US Government agencies, threaten health systems and could lead to an increase in avoidable deaths in SSA [22]. This underscores the urgent need for innovative financing to expand domestic resources for health and improve spending efficiency amid shrinking budgets. Although it cannot replace the ongoing donor shortfall, the elimination of ghost workers can play an important role in increasing resources for sustainable financing.

## Eliminating ghost workers and seeking efficiency to optimize resources

Addressing the contradiction of an unpaid and underutilized community health workforce alongside ghost workers requires comprehensive action. Addressing the ghost worker challenge is crucial to enhancing efficiency and maximizing the utilization of scarce resources. Governments should begin by conducting thorough audits and staff verification to remove ghost workers from public payrolls. Previous efforts highlight how this step has unlocked substantial funds and, as a result, led to health worker motivation and service delivery improvement [16]. For example, Nigeria identified about 65,000 ghost workers through the implementation of the Integrated Payroll and Personnel Information System from 2007 to 2015, generating a total saving of $1.1 billion [16]. The Democratic Republic of Congo saves an estimated $1.8 million annually by identifying ghost workers through its Human Resource Information System [16]. In Mozambique, a nationwide “proof of life” audit conducted from 2015 to 2017 identified 30,000 ghost workers, generating savings of $250 million [16]. In 2017, Ghana removed 26,589 ghost workers from the government payroll, saving $103 million per year [17]. Moreover, in the Central African Republic, the Ministry of Public Service and Administrative Reform carried out a physical verification of civil servants in 2022. The review uncovered several irregularities, including ghost employees, double counts, staff outside legal frameworks or with multiple ID numbers, and salary discrepancies between payroll records and personnel files. The review identified and removed 2,871 ghost workers, resulting in monthly salary savings of USD 1.798 million [23]. A similar pattern is seen in East and Southern Africa, where the Tanzanian government identified 16,137 fictitious workers in August 2016, saving a total of 6.9 million USD per month [17]. Additionally, the Zimbabwe government, through a biometric exercise, removed 10,000 ghost workers in 2020 who had been diverting significant public resources [17].

## Reinvesting optimized resources in primary healthcare

The substantial savings achieved through eliminating ghost workers and implementing efficiency reforms create a valuable opportunity. Strategically re-investing these domestically generated resources into primary healthcare, especially prioritizing the integration of CHWs into the civil service system, is paramount. Formally integrating professional CHWs into the civil service aligns with WHO’s recommendation to remunerate them with a financial package commensurate with their job demands, complexity, working hours, training, and responsibilities [8]. Moreover, an evaluation of legal frameworks for CHW compensation in five countries found that public sector employment models effectively support WHO recommendations by providing formal, paid, and fair contracts that meet international labor standards [24]. At the same time, resources gained from efficiency reforms can also be used to enhance the motivation and retention of CHWs through standardized training programs and clear career pathways [8,24,25].

Compensation schemes that place CHWs on government payroll are particularly effective, especially in ensuring sustainable community health programs [26,27]. Liberia provides a recent example of comprehensive civil service reform, including the audit of personal records, the institution of an electronic recruitment system, and the strengthening of transparency and accountability in the use of public funds. Accordingly, an audit across 103 government entities eliminated 5,536 ghost workers, saving an estimated $1.8 million annually. Additionally, measures to regulate expenditure on consultancy recruitment saved $4.6 million in 2024. Implementing fraud prevention measures for mobile money salary payments saved an additional $1.85 million. Collectively, these reforms generated approximately $8.3 million in 2024 alone [28]. The reform in Liberia created an opportunity for young graduates to join the civil service, including the integration of professional CHWs into the civil service system with a monthly salary [28]. The reform in Liberia also serves as a model by leveraging the resources freed to transition 300 Community Health Service Supervisor positions from donor support into the civil service. This approach will enhance CHWs’ motivation and performance in Liberia by integrating them into the civil service system. It will also support the sustainability of the national community health program and provide a valuable example for other countries.

## Multisectoral collaborative action for sustainable solutions

Strengthening multisectoral collaborative action is essential. Ministries of health, finance, and civil service commissions need to coordinate closely, while civil society and independent auditors ensure transparency and accountability [29] (see Box 1). The Health and Public Service Network of Africa (HaPSNA) is a regional platform of Ministries of Health and Civil Service Commissions, launched to collectively address the region’s human health workforce challenges. The Network aims to create opportunities for knowledge exchange and peer learning among countries by acknowledging regional efforts to address pressing challenges of the human health workforce in the region, including ghost workers, absenteeism, and performance management. The priorities of HaPSNA include improving workforce governance, coordination, and efficiency in health and public service delivery among the collaborating countries. It brings experts and policymakers from the Ministry of Health and civil service agencies to drive meaningful reforms, including the integration of CHWs into the civil service system. Based on the spirit of South–South partnership—a strategic and ongoing collaboration between two or more countries in the global south that pursue shared development goals and mutually benefit each other outcomes-the network members developed a Community Health Program Maturity Matrix and Index [30]. The tool enables countries self-assess how well community health programs are embedded in primary healthcare and civil service systems, and to identify areas for improvement.

**Box 1 Key actions to enable civil service reforms that benefit the human health workforce, including the integration of CHWs into the civil service**

- Conduct civil service reform through a comprehensive audit of personnel records across government entities with standardized employment documentation and promote merit-based recruitment;
- Identify “low-hanging” areas within the civil service that are characterized by wasteful inefficiencies and lack of transparency to enhance accountability and effective use of public funds;
- Set up an independent body that lets civil servants challenge unfair job decisions and safely report wrongdoing without retaliation;
- Eliminate paper-based recruitment systems in the public sector and modernize it with the use of advanced technology and a meritocratic foundation;
- Reinvest the resources gained from addressing inefficiencies into strengthening primary healthcare and CHW programs including integrating CHWs into the civil service;
- Strengthen cross-sectoral collaboration between Ministries of Health, Civil Service Agencies, Ministries of Finance, and key stakeholders to institutionalize sustainable measures to strengthen the human health workforce;
- Leverage the exchange of knowledge and best practices to advance peer learning among countries sharing the same challenges across the human health workforce.

Global and regional health workforce platforms, such as WHO-led Global Health Workforce Alliance and a network of Human Resource for Health (HRH) managers from the Ministry of Health in nine Francophone African countries, address similar HRH challenges without focused engagement of civil service agencies [31,32]. However, HaPSNA’s experience of primarily engaging with civil service agencies is well-positioned to address cross-cutting health workforce issues in a multisectoral manner.

## Conclusions

Enhanced fiscal accountability and allocating public resources effectively for health workforce development require targeted reforms to address public sector inefficiencies, including ghost workers and payroll inefficiencies, through coordinated multisectoral action. Several African countries have shown that civil service reforms targeting such inefficiencies can optimize resources. Liberia’s recent reinvestment of savings from civil service reforms to integrate CHWs into the public service stands as a commendable example. Redirecting resources recovered from ghost workers offers an important opportunity to finance CHWs, but it should be regarded as a complementary mechanism for sustainable financing. Countries must strengthen resource mobilization and diversify domestic funding to finance CHWs. At the same time, leveraging intercountry and regional partnerships to exchange proven strategies and accelerate the formal integration of professional CHWs into the civil service and the national health systems. To ensure the sustainability of these reforms, engaging local communities, health workers, and policymakers is essential, supported by a monitoring and evaluation framework to measure the reforms’ impact on health outcomes and workforce efficiency. The establishment of HaPSNA creates an opportunity for peer and collaborative learning among African countries to address health workforce challenges through South-South partnerships and collaboration between Ministries of Health and Civil Service. The development of the maturity matrix is a significant milestone to measure progress through self-assessment and peer evaluation of the integration of CHWs into the civil service. Such efforts surpass matters of justice for frontline workers, but are a key objective for building health systems capable of delivering on the promise of UHC.

## Abbreviations

CHWs: Community Health Workers
HaPSNA: Health and Public Service Network of Africa
HRH: Human Resource for Health
LMICs: low- and middle-income countries
SSA: sub-Saharan Africa
UHC: Universal Health Coverage
WHO: World Health Organization
